# Computational and experimental analyses of retrotransposon-associated minisatellite DNAs in the soybean genome

**DOI:** 10.1186/1471-2105-13-S2-S13

**Published:** 2012-03-13

**Authors:** Lauren S Mogil, Kamil Slowikowski, Howard M Laten

**Affiliations:** 1 Program in Bioinformatics Loyola University Chicago, 1032 W. Sheridan Rd, Chicago, IL 60660 USA; 2 Department of Biology, Loyola University Chicago, 1032 W. Sheridan Rd, Chicago, IL 60660 USA; 3 Present address: Department of Biochemistry and Molecular Biology, Mayo Graduate School, Rochester, MN 55905 USA

## Abstract

**Background:**

Retrotransposons are mobile DNA elements that spread through genomes via the action of element-encoded reverse transcriptases. They are ubiquitous constituents of most eukaryotic genomes, especially those of higher plants. The pericentromeric regions of soybean (*Glycine max*) chromosomes contain >3,200 intact copies of the Gmr9/GmOgre retrotransposon. Between the 3' end of the coding region and the long terminal repeat, this retrotransposon family contains a polymorphic minisatellite region composed of five distinct, interleaved minisatellite families. To better understand the possible role and origin of retrotransposon-associated minisatellites, a computational project to map and physically characterize all members of these families in the *G. max *genome, irrespective of their association with Gmr9, was undertaken.

**Methods:**

A computational pipeline was developed to map and analyze the organization and distribution of five Gmr9-associated minisatellites throughout the soybean genome. Polymerase chain reaction amplifications were used to experimentally assess the computational outputs.

**Results:**

A total of 63,841 copies of Gmr9-associated minisatellites were recovered from the assembled *G. max *genome. Ninety percent were associated with Gmr9, an additional 9% with other annotated retrotransposons, and 1% with uncharacterized repetitive DNAs. Monomers were tandemly interleaved and repeated up to 149 times per locus.

**Conclusions:**

The computational pipeline enabled a fast, accurate, and detailed characterization of known minisatellites in a large, downloaded DNA database, and PCR amplification supported the general organization of these arrays.

## Background

The genomic landscapes of most higher eukaryotes are dominated by repetitive DNAs [1-3]. Most genome-wide, interspersed repeats are retrotransposons, including long and short interspersed elements (LINEs and SINEs, respectively) and long terminal repeat (LTR) retrotransposons [1,3]. The action of LINE- or LTR retrotransposon-encoded reverse transcriptases on transcribed RNA intermediates and integration of the resulting cDNAs has resulted in the accumulation of thousands of these elements dispersed throughout the genomes of nearly all eukaryotic species [1,3].

**Figure 1:**
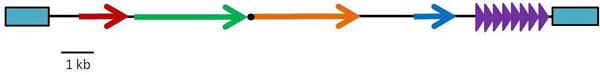
**Structure of Gmr9/GmOgre retrotransposon**. Blue blocks represent the LTRs; the red arrow represents ORF1 (protein of unknown function); the green arrow represents *gag-pol *exon 1, the orange arrow represents *gag-pol *exon 2; the blue arrow represents ORF 3 (protein of unknown function); the black dot represents the *gag-pol *intron; the stacked purple arrows represent the minisatellite array.

LTR retrotransposons range in length from a few hundred base pairs (non-autonomous, truncated copies) to >25,000 bp [3]. Most autonomous elements encode structural proteins (gag) that assemble into intracellular virus-like particles, and enzymes (pol) required for polyprotein processing, reverse transcription, and cDNA integration (Figure 1) [3]. Most elements are littered with incapacitating mutations, including large insertions and deletions [1,3].

The proliferation of retrotransposons can be highly disruptive to gene and genome structure and function, and host mechanisms can silence and eliminate elements [4,5]. However, there is increasing evidence that retrotransposons have made important contributions to the evolution of gene and genome structure and function [6].

One feature of a few of these LTR retroelements is the presence of other classes of repeats within their DNA, specifically microsatellites and minisatellites [7-10]. Gmr9/GmOgre from soybean (Figure 1) is an uncharacteristically long and relatively high copy-number retrotransposon with a canonical representative >21 kb in length and in excess of 3,200 copies per genome [11,12]. A member of the Ty3-gypsy retrotransposon superfamily, most copies are restricted to pericentromeric regions of all twenty soybean chromosomes [11]. Members of this family and related elements in other plant species contain a polymorphic minisatellite (MS) array of several hundred base pairs just downstream of the coding region [7,12,13]. A combination of computational and experimental approaches was used to map and fully characterize the organization and distribution of the five Gmr9-associated MS throughout the soybean genome.

## Methods

### Computational methods

All *G. max *assembled chromosome sequences [14] were downloaded from GenBank and made into a BLAST database. Details and implementation of the computational pipeline are described in Note 1 in Additional file 1 and is available at the link https://github.com/slowkow/soy-rtms.

### Experimental methods

Genomic DNA was isolated using a DNeasy Plant Mini Kit (Qiagen) from 100 mg of leaf tissue from *Glycine max *cv Williams 82 ground to a fine powder under liquid nitrogen. Primer sequences and cycling parameters are described in Note 2 in Additional file 1.

## Results

### Computational analysis and results

The Gmr9/GmOgre MS region has five distinct repeat families designated A through E. The consensus sequences have been reported [12,15-19]. The lengths were 26, 38, 37, 105 and 43 bp, respectively (see Note 3 in Additional file 1). Nine of the last 11 bp of repeats B and C are identical, and could be considered sub-repeats, but otherwise there are no detectable sequence similarities among any of the repeat families. BLASTn searches of all Genbank DNA databases, from which *Glycine *sequences were excluded, retrieved no similar sequences (see Note 4 in Additional file 1).

**Figure 2:**
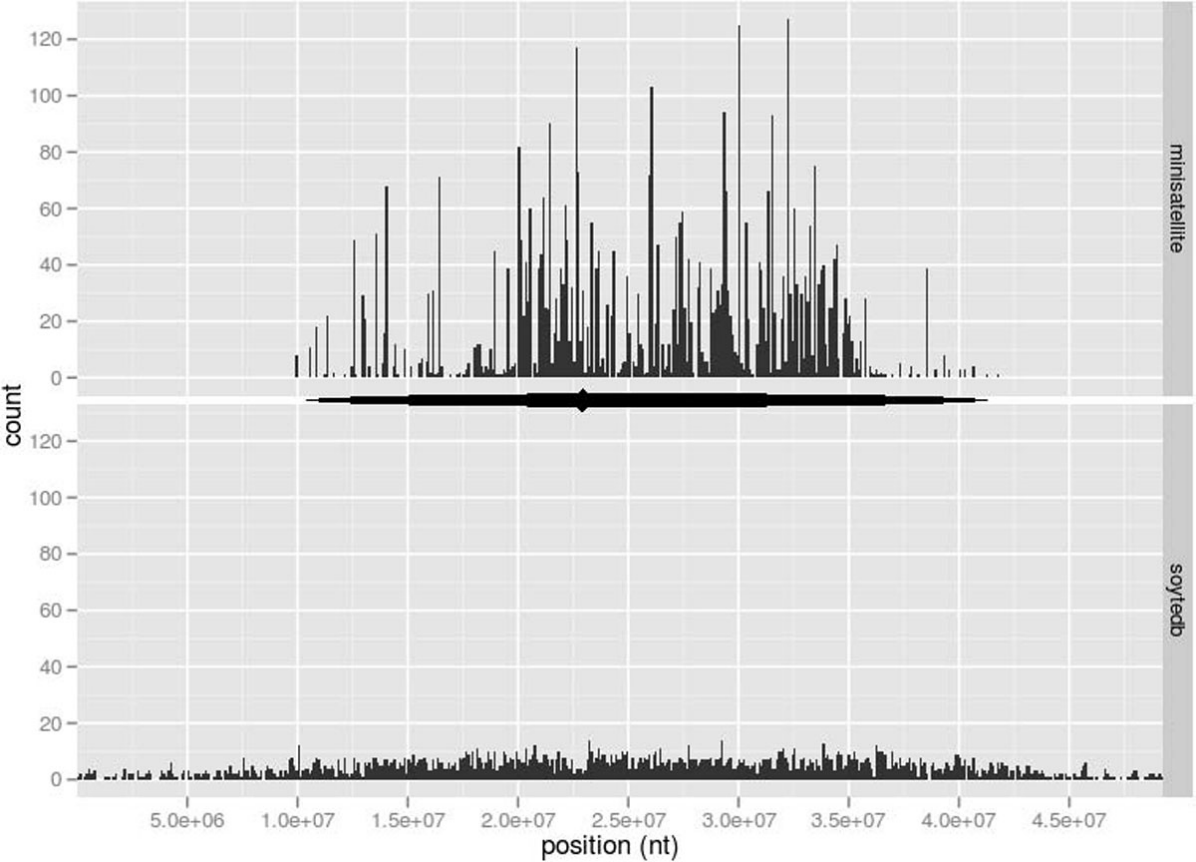
**Density distributions of TE and MS on *G. max *Chromosome 4**. MS sequences A through E (top panel) and TE (bottom panel) per 100,000 bp. Horizontal lines between panels represent locations of the pericentromeric region and the putative centromere (◆) from [29].

Individual queries of the five MS consensus sequences against the downloaded soybean chromosome database resulting in 63,841 unique hits with ≥90% identity, of which 51,154 (80%) were within the map coordinates of annotated retrotransposons (Table 1 and Figure 2). Of these, a total of 40,150 (78%) fall within the coordinates of an "intact" member of the Gmr9 family (Table 1). In addition to Gmr9, 42 other defined retrotransposon families representing both Ty3-gypsy and Ty1-copia superfamilies contain at least one of the MS sequences (Table 1). With the exception of Gmr5 and Gmr6, the MS repeats were generally more plentiful among Ty3-gypsy superfamily members than Ty1-copia members (Table 1).

**Table 1:** Distribution of MS repeats among retrotransposons and TE's

Family^1^	Super-family	No. of intact copies^1^	Repeat A		Repeat B		Repeat C		Repeat D		Repeat E		Additional discreet loci^2^
			Total hits	No. of discreet loci	Total hits	No. of discreet loci	Total hits	No. of discreet loci	Total hits	No. of discreet loci	Total hits	No. of discreet loci	
Gmr1	Gypsy	564	121	15	67	14	70	15	28	15	49	16	16
Gmr2	Copia	837	24	5	34	5	37	5	8	4	17	6	4
Gmr3	Gypsy	867	88	12	72	10	87	13	15	8	33	12	28
Gmr4	Gypsy	1363	354	38	224	37	226	38	90	38	150	50	78
Gmr5	Copia	401	141	20	73	13	75	14	28	14	50	18	42
Gmr6	Copia	763	203	19	115	18	141	23	41	18	68	20	12
Gmr7	Copia	195	10	1	4	1	6	2	3	2	6	3	1
Gmr9	Gypsy	3247	13293	1561	9137	1428	9211	1543	2999	1468	5510	1890	3975
Gmr12	Gypsy	114	44	5	34	5	42	5	10	3	20	6	2
Gmr14	Copia	43	0	0	0	0	1	1	0	0	0	0	0
Gmr15	Copia	84	4	1	4	1	1	1	1	1	2	1	0
Gmr16	Copia	116	6	1	0	0	0	0	0	0	0	0	0
Gmr17	Gypsy	422	23	3	16	2	2	2	2	2	4	2	1
Gmr18	Copia	204	10	2	14	2	16	2	5	2	8	3	1
Gmr19	Gypsy	581	214	27	113	24	114	22	46	20	76	28	19
Gmr21	Gypsy	157	85	12	89	14	76	13	34	15	59	18	6
Gmr22	Copia	119	3	1	4	1	1	1	1	1	2	1	0
Gmr24	Copia	79	2	1	5	1	4	1	0	0	2	1	0
Gmr25	Gypsy	307	111	13	61	11	71	13	28	10	48	13	21
Gmr28	Copia	100	7	1	14	1	15	1	2	1	4	1	0
Gmr34	Gypsy	120	2	1	4	1	1	1	1	1	2	1	9
Gmr35	Copia	95	9	1	8	1	11	2	2	1	4	1	0
Gmr37	Copia	255	35	4	50	5	51	5	9	4	22	10	5
Gmr51	Gypsy	27	4	1	0	0	0	0	1	1	3	2	0
Gmr52	Gypsy	20	6	1	0	0	0	0	0	0	0	0	0
Gmr59	Gypsy	31	0	0	0	0	3	1	0	0	0	0	0
Gmr68	Copia	12	13	1	6	1	7	1	3	1	6	2	0
Gmr75	Gypsy	17	9	2	6	2	5	2	4	2	6	2	0
Gmr79	Gypsy	49	3	1	0	0	2	1	0	0	0	0	0
Gmr80	Gypsy	6	12	1	5	1	7	1	2	1	4	1	0
Gmr90	Copia	10	9	1	4	1	5	1	4	1	5	1	0
Gmr110	Gypsy	6	0	0	0	0	2	1	0	0	0	0	0
Gmr123	Gypsy	3	0	0	0	0	1	1	0	0	0	0	0
Gmr128	Gypsy	5	10	2	7	2	9	2	5	2	8	2	0
Gmr139	Gypsy	88	28	4	26	4	14	4	5	4	11	4	12
Gmr146	Gypsy	3	0	0	0	0	0	0	0	0	1	1	0
Gmr163	Gypsy	3	1	1	5	1	6	1	2	1	4	1	0
Gmr169	Gypsy	18	12	1	5	1	8	2	4	3	10	4	4
Gmr190	Copia	21	12	1	5	1	7	1	3	2	7	2	0
Gmr192	Gypsy	3	0	0	0	0	0	0	0	0	1	1	0
Gmr290	Gypsy	3	1	1	0	0	0	0	1	1	0	0	0
Gmr459	Gypsy	10	0	0	0	0	0	0	0	0	1	1	0
Gmr522	Gypsy	17	6	1	6	1	0	0	1	1	2	1	0
Others^2^	Multi	ND	ND	ND	ND	ND	ND	ND	ND	ND	ND	ND	17
Unknown	NA	NA	ND	ND	ND	ND	ND	ND	ND	ND	ND	ND	75
Total		11385	14915	1763	10217	1610	10335	1742	3388	1648	6205	2126	4328

ND: Not determined; NA: Not applicable

^1^From Du et al. [11]

^2^From unannotated sites (see text); includes 9 LTR retrotransposons, 1 LINE, and 7 DNA transposons

The remaining 18,781 MS hits fell outside of annotated transposable elements (TE) and clustered into a total of 4,328 loci. Ninety-two percent of the DNA sequences (3,975) were at least 80% identical over a length of **≥**400 bp to annotated copies of Gmr9 found elsewhere in the genome (Table 1). This far exceeded the number of discreet MS hits initially found for Gmr9, as did the corresponding data for Gmr3, Gmr4, Gmr5, Gmr25, and Gmr139. Of the remaining 354 unannotated loci, all but 75 could be assigned to a TE family. DNA's from the unidentified 75 loci were queried against the nr and gss Genbank databases and all retrieved >25 hits with e values <10^-10 ^in one or both of these databases, indicating that all were repetitive families. No further analyses of these sequences were undertaken (see Note 5 in Additional file 1).

**Figure 3:**
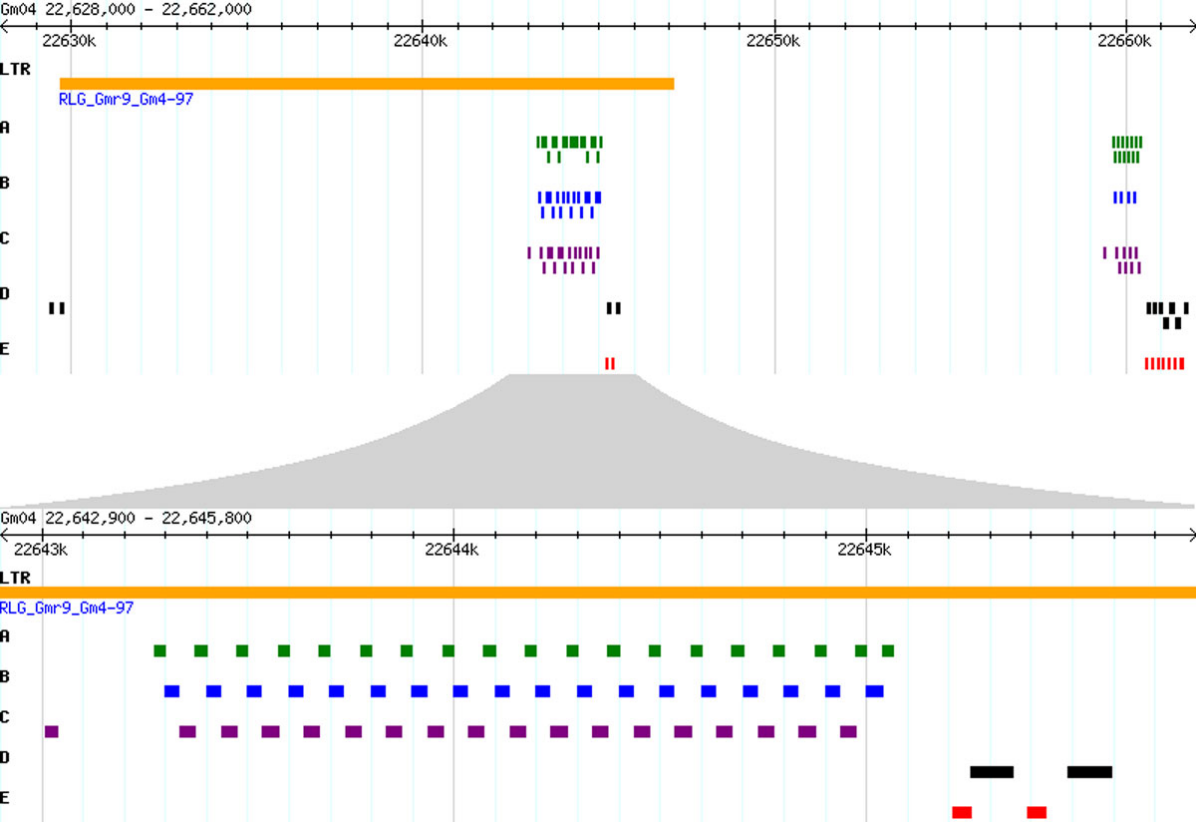
**Organization of MS sequences within copies of Gmr9 on chromosome 4**. Top: Full length Gmr9_Gm4-29 with an adjacent Gmr9 member with MS region to the right. Bottom: Gmr9_Gm4-97 MS region showing alternating MS sequences.

The average number of repeats per Gmr9 element - the ratio of total hits to discreet hits - was 8.5 for repeat A, 6.4 for repeat B, 6.0 for repeat C, 2.0 for repeat D, and 2.9 for repeat E. These values were consistent with the organization of the consensus sequence reported previously [12]. The total number of hits was considerably smaller for most of the other families. Figure 2 illustrates the distribution and density of TE and the five MS on chromosome 4. The densities of MS and TE are strongly correlated, and the former are restricted to the pericentromeric region. Figure 3 represents a 34 kb section of Chromosome 4 with two tandem Gmr9 family members (top) and an expanded region of 2.9 kb from Gmr9_Gm4-9 (bottom). The MS array extends across 2.6 kb and consists of 17 tandem repeats of A-B-C, followed by one tandem array of A-B-A. Approximately 120 bp downstream of the last A repeat there is one D-E repeat followed by a break of about 100 bp and a second E repeat.

[ABAC]_n _was the primary pattern found in the MS arrays, but other arrays of [ABC]_n _as found for Gm4-97 (Figure 3) and [ACB]_n _were retrieved (Table S1 in Additional file 2). The longest unbroken tandem array consisted of 37 repeats of ABAC. The total length of this array was 4,760 bp. Other long, unbroken tandem arrays were found in which ABC was repeated 16 to 28 times to total lengths of nearly 3,000 bp. The longest unbroken tandem array of ACB was nearly 1,800 bp in length. The majority of arrays were far shorter (see Table S1 in Additional file 2 and Note 6 in Additional file 1).

Of the approximately 22,500 copies of repeat A retrieved, nearly 75% were identical to the consensus sequence, and another 20% differed by a single base pair (Fig. S1 in Additional file 3). In the case of repeat B, almost 44% of the approximately 17,650 copies of this repeat were identical to the consensus with the remaining 56% distributed among several different variants (Fig. S1). Repeat D, the longest repeat, was far more polymorphic than the other repeats, with a greater number of sequences that varied significantly from the consensus in identity and length (Figs. S1 and S2 in Additional file 3). Length variants of the other repeats are shown in Fig. S2 (see Note 7 in Additional file 1). Repeat A has virtually no length variants.

### PCR results

Electrophoretic separation of the amplification products generated from all primer combinations resulted in long ladders of closely spaced bands (Fig. S3 in Additional file 3). The longest amplicons were in excess of 3 kb, consistent with the computational findings (see Table S1).

## Discussion and conclusions

Gmr9/GmOgre is one of a number of plant retrotransposons in the Ogre retrotransposon lineage that contain embedded satellites (see Note 8 in Additional file 1) [7,12,13]. In the case of the five MS families initially found in Gmr9, we have shown that every single copy is embedded in a repetitive DNA, 99% of which are LTR retrotransposons, and most of these are Gmr9 copies (see Note 9 in Additional file 1). Virtually all are found in pericentromeric regions of all twenty *G. max *chromosomes. The origin of the MS repeats is clearly Gmr9, but the means by which other retrotransposon families acquired them is unknown.

The considerable repeat number variation among the clusters of MS loci (Table S1) was not unexpected. The mechanisms sponsoring expansions and contractions of satellite repeats, including polymerase slippage, gene conversion, non-allelic homologous recombination, and post-replicative DNA repair [2], might be elevated for several reasons. For instance, in the case of slippage, host RNA polymerase, element-encoded reverse transcriptase, and host DNA polymerase could all contribute. The sheer number of retrotransposon loci carrying these MS clusters creates thousands of potential sites for non-allelic recombination. The maintenance of the relatively high sequence identity of repeats A, B, and C suggests that gene conversion may be homogenizing these sequences.

The possible functions, if any, of these MS sequences reported here are not known. These and other more distantly related retrotransposons that possess internal MS regions [20-23] invite speculation about the origins and possible functions of these DNAs. Pericentromeric regions are highly enriched for both retrotransposons and centromere-specific MS DNAs and both classes are recovered in centromere-specific histone H3 chromatin immunoprecipitation assays [24-27]. Alternatively, centromeric retrotransposons may contribute to molecular processes that facilitate the formation of centromeric chromatin [28]. Minisatellites embedded in mobile elements that target centromeres would be an effective pairing for the dispersal and amplification of sequences that contribute to centromere function.

Computational tools enabled a complete physical characterization of the polymorphisms, map positions, and organization of five MS in the soybean genome. The results confirm that these particular MS are universally embedded in other repetitive DNA classes, primarily LTR retrotransposons, the majority of which are members of the Gmr9 retrotransposon family.

## Competing interests

The authors declare that they have no competing interests.

## Authors' contributions

LSM carried out the experimental work and contributed to general design, implementation, and analysis of computational outputs. KS designed, developed, and implemented all computational tools and contributed to analysis of outputs. LSM and KS contributed to the initial draft of the manuscript. HML conceived of the study, participated in its design and coordination, and generated the final draft of the manuscript. All authors read and approved the final manuscript.

## Supplementary Material

Additional file 1 **Mogil_Additional_file_1.pdf contains supplemental text notes 1 through 9 referenced in the main text, and additional references**. Click here for file

Additonal file 2 **Mogil_Additional_file_2.xls contains Table S1 that provides a detailed listing of all extended microsatellite patterns**. Click here for file

Additional file 3 **Mogil_Additional_file_3.pdf contains Figures S1 and S2 that depict sequence identity and sequence length hisograms, respectively, and Figure S3 which is a photograph of an ethidium bromide-stained gel of PCR products**. Click here for file
