# Supplementary material for: Computational and experimental analyses of retrotransposon-associated minisatellite DNAs in the soybean genome
Source: BMC Bioinformatics. 2012 Mar 13;13(Suppl 2):S13. doi: 10.1186/1471-2105-13-S2-S13 (PMC3305785; doi:10.1186/1471-2105-13-S2-S13)
Supplement: Additional file 1 — Mogil_Additional_file_1.pdf contains supplemental text notes 1 through 9 referenced in the main text, and additional references. [file 1471-2105-13-S2-S13-S1.pdf]

Additional File 1

Note 1: Two hundred members of each of the five MS repeats [1] were retrieved from this database by BLASTn searches [2] and a strict consensus was generated for each set using Geneious 5.4 (Biomatters). 38,582 TE annotations and the corresponding genome coordinates were downloaded from SoyTEdb, a database of *G. max* transposable elements [3]. The five MS sequences were queried against the local soybean chromosome database. BEDTools [4] and custom Perl scripts [5] were used to count overlaps between annotations and BLAST hits and to analyze results, and GNU parallel [6] was used to accelerate part of the analysis (see Additional File 2). Two windows of 500 bp each upstream and downstream of any MS were used to define whether or not any clusters of MS were in the same or in different loci. BLASTn searches using each of the five MS sequences as queries were performed against all Genbank DNA databases. Figures were generated as described in Additional File 2 [7].

Note 2: Forward and reverse primers for polymerase chain reaction (PCR) amplification for selected repeat families were chosen using Netprimer (Premier Biosoft) and purchased from Eurofins MWG Operon. The primers used were: Bfor: CAAGCAGAGACCAAGTTTGG; Brev: TACAAGGGTTGCAGAATGACC; Cfor: GTTATCCGCACCCTTTTTG; Crev: AACCGTAAGGTATCTCCGC; Dfor: GTCAACCGAGAGGAGCGAA; Drev: CACAAATTGCCTAATGATGGG. Repeat A did not contain any sub-sequences that would serve as suitable PCR primers. PCR amplifications were run in volumes of 25µl with GoTaq Green (Promega), 200 ng of template DNA, and the following primer pairs at concentrations of 1µM each: Bfor/Brev, Cfor/Crev, Dfor/Drev, Bfor/Crev, and Cfor/Brev. Thirty cycles were run with denaturation at 95ºC, annealing at 53ºC and extension at 72 ºC. Extension reactions were run for 30 seconds with a final extension of 5 minutes. PCR products were electrophoresed on a 1.5% agarose gel and stained with ethidium bromide.

Note 3: The initial generation of a Gmr9 consensus assembly resulted in a sequence of eight tandem arrays of ABAC followed by three tandem arrays of DE [1], although at the time of publication, D [8] and E [9] were considered to be a single, fused repeat. The two arrays were separated by approximately 60 bp [1].

Note 4: BLASTn searches using the five MS sequences in queries of all Genbank DNA databases, from which *Glycine* sequences were excluded, retrieved no similar sequences, with the exception of likely database submission errors. The exceptions, which we presumed to be mishandling or mislabelling errors because the sequences were 100% identical to soybean copies of all five repeats, were *Oryza australiensis* (rice) and *Phaseolus vulgaris* (bean) accessions in the Genbank Genome Survey Sequence database. The labs working on these genome projects were simultaneously sequencing the *G. max* genome.

Note 5: In addition to hits on intact elements, a much smaller number of hits described as solo LTRs [3] were also found (data not shown). Solo LTRs are generated by intra- and inter-element recombination between the LTRs of a single element or nearby paralogs, respectively, that lead to the complete deletion of the intervening DNA [10]. Examination of randomly selected hits to sequences labeled as solo LTRs in the SoyTE database [3] revealed that these sequences contained non-LTR extensions into upstream retrotransposon DNA and that the MS hits were in these extensions. Thus, it is unlikely that any of these MS sequences are in LTRs. Because of the fragmentation of older retrotransposon insertions and the frequency of nested insertions, it is possible that the other retrotransposons contain vestiges of Gmr9. However, the SoyTE database

enables the identification of nested insertions [3], and none of the MS hits assigned to other retrotransposons in Table 1 were found to be in nested copies of Gmr9.

Note 6: Most of the arrays recovered contained fewer than 20 tandem monomers and are not included in Table S1 because they do not provide useful information. Most of these shorter arrays are part of longer clusters but were not included in the output due to sequence heterogeneity, especially in repeats B, C, D, and E. The computational algorithm used restricted the tandem array analyses to repeats that were separated from adjacent repeats by ≤5 bp, with high sequence identity to the corresponding consensus sequence (see below). This was especially relevant to finding long arrays containing tandem copies of D or E, and no tandem arrays longer than 4 monomers were retrieved. While the longest tandem arrays tended to contain the same order of repeats, other long tandem repeats were mixtures of patterns and or interspersed with shorter runs of two to five repeats of AC. Most of the arrays recovered contained fewer than 20 tandem monomers and are not included in Table S1 because they do not provide useful information. Most of these shorter arrays are part of longer clusters but were not included in the output due to sequence heterogeneity, especially in repeats B, C, D, and E. The computational algorithm used restricted the tandem array analyses to repeats that were separated from adjacent repeats by ≤5 bp, with high sequence identity to the corresponding consensus sequence. This was especially relevant to finding long arrays containing tandem copies of D or E, and no tandem arrays longer than 4 monomers were retrieved.

Note 7: For repeats B and C, there were two major forms that made up ~50% of the sequences, and considerable length variation at the T-rich 3’ end of C involving the last 7 base pairs (Figs. S1 and S2). This is the region of sequence similarity repeat C shares with repeat B (see above). Repeats D and E were far more polymorphic than the other repeats with a greater number of sequences that varied significantly from the corresponding consensuses in both identity and length (Figs. S1 and S2). Repeat D had approximately equal numbers of a 105-bp sequence and one with a 15-bp 3’ end extension, and there were three discreet classes of truncated repeat D copies (Fig. S2). There was virtually no length polymorphism in the A or E families (Fig. S2).

Note 8: Despite the clear evolutionary relationships among members of the Ogre lineage and the retention of several structural characteristics unique to the lineage, including two homologous open reading frames of unknown function and a conserved intron [11], the MS sequences are not detectably similar despite the conservation of their location.

Note 9: Based on the ratio of discreet D hits to the number of annotated TE in the SoyTEdb (Table 1), nearly 60% of the members of Gmr9 contain a MS array. This is likely to be an underestimate of the true proportion. MS sequences with less than 90% identity were initially not included, and it was not determined if the full constellation of MS sequences were present in every one of the Gmr9 elements in Table 1. Because the lengths of “intact” Gmr9’s range from under 8,000 to over 26,000 bp [3], it is likely that most if not all of the Gmr9’s lacking MS copies resulted from long deletions that encompassed the MS region in the shortened elements.

References

1. Laten HM, Mogil LS, Wright LN: **A shotgun approach to discovering and reconstructing consensus retrotransposons ex novo from dense contigs of short sequences derived from Genbank Genome Survey Sequence database records**. *Gene* 2009, **448:**168-173.

2. Zhang Z, Schwartz S, Wagner L, Miller W: **A greedy algorithm for aligning DNA sequences**. *J Comput Biol* 2000, **7:**203-214.
3. Du J, Grant D, Tian Z, Nelson RT, Zhu L, Shoemaker RC, Ma J: **SoyTEdb: a comprehensive database of transposable elements in the soybean genome**. *BMC Genomics* 2010, **11:**113.
4. Quinlan AR, Hall IM: **BEDTools: a flexible suite of utilities for comparing genomic features**. *Bioinformatics* 2010, **26:**841-842.
5. Stajich JE, Block D, Boulez K, Brenner SE, Chervitz SA, Dagdigian C, Fuellen G, Gilbert JG, Korf I, Lapp H, Lehvaslaiho H, Matsalla C, Mungall CJ, Osborne BI, Pocock MR, Schattner P, Senger M, Stein LD, Stupka E, Wilkinson MD, Birney E: **The Bioperl toolkit: Perl modules for the life sciences**. *Genome Res* 2002, **12:**1611-1618.
6. Tange O: **GNU Parallel - The Command-Line Power Tool**. *;login: The USENIX Magazine* 2011, **36:**42-47.
7. Wickham H: *ggplot2: Elegant Graphics for Data Analysis.* New York: Springer; 2009.
8. Mogil, LS, Laten HM: **Retrotransposon-associated minisatellites in Glycine max: MSAT-105_Gm**. *Repbase Rep* 2010, **10:**2178.
9. Mogil LS, Laten HM: **Retrotransposon-associated minisatellites in Glycine max: SAT-43_Gm**. *Repbase Rep* 2011, **11:**in press.
10. Vitte C, Panaud O: **LTR retrotransposons and flowering plant genome size: emergence of the increase/decrease model.** *Cytogenet Genome Res* 2005, **110:**91-107.
11. Macas J, Neumann P: **Ogre elements--a distinct group of plant Ty3/gypsy-like retrotransposons**. *Gene* 2007, **390:**108-116.
