# Supplementary material for: Computational and experimental analyses of retrotransposon-associated minisatellite DNAs in the soybean genome
Source: BMC Bioinformatics. 2012 Mar 13;13(Suppl 2):S13. doi: 10.1186/1471-2105-13-S2-S13 (PMC3305785; doi:10.1186/1471-2105-13-S2-S13)
Supplement: Additional file 3 — Mogil_Additional_file_3.pdf contains Figures S1 and S2 that depict sequence identity and sequence length hisograms, respectively, and Figure S3 which is a photograph of an ethidium bromide-stained gel of PCR products. [file 1471-2105-13-S2-S13-S3.pdf]

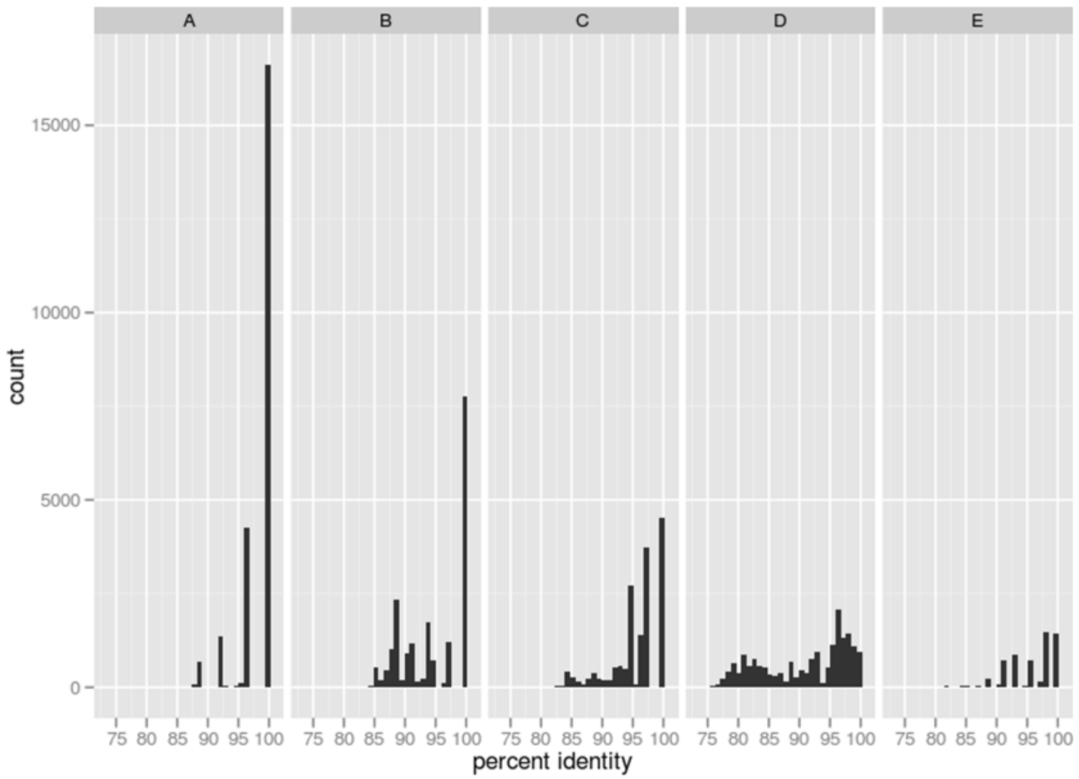

Figure S1. Sequence identity histograms of MS repeats A through E relative to consensus sequences.

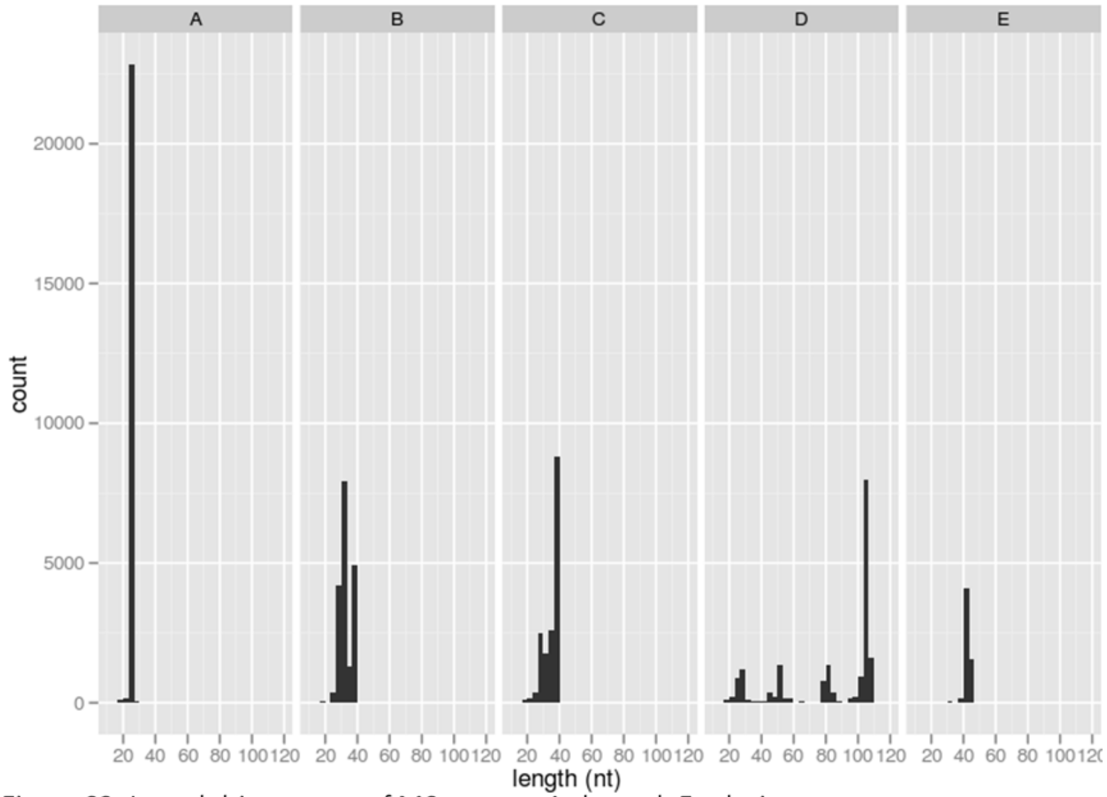

Figure S2. Length histograms of MS repeats A through E relative to consensus sequences. Length display bars reflect the assignment of all repeats to size bins in three-nucleotide increments.

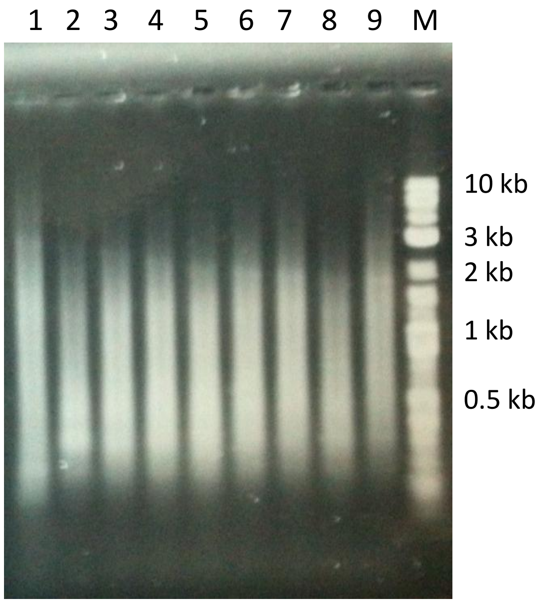

Figure S3. Ethidium-bromide stained agarose gel of PCR amplification products. Lanes 1 and 2: Bfor/Brev; lanes 3 and 4: Cfor/Crev; lane 5 and 6: Bfor/Crev; lanes 7 and 8: Cfor/ Brev; lane 9: Dfor/Drev; lane M: 2-log DNA ladder.
